# Steroids' Neuroprotective Potential in Severe Cerebral Venous Thrombosis: Experimental and Clinical Exploration of NLRP3 Inflammasome Inhibition

**DOI:** 10.1111/cns.70125

**Published:** 2024-11-17

**Authors:** Shuyuan Hu, Yaqin Gu, Limin Hou, Jia Liu, Haiping Zhao, Yumin Luo, Chunxiu Wang, Xunming Ji, Guiyou Liu, Jiangang Duan

**Affiliations:** ^1^ Department of Emergency Xuanwu Hospital Capital Medical University Beijing China; ^2^ Neurology and Intracranial Hypertension & Cerebral Venous Disease Center, National Health Commission of China, Xuanwu Hospital Capital Medical University Beijing China; ^3^ Beijing Institute of Brain Disorders, Laboratory of Brain Disorders, Ministry of Science and Technology, Collaborative Innovation Center for Brain Disorders Capital Medical University Beijing China; ^4^ Cerebrovascular Diseases Research Institute and Department of Neurology, Xuanwu Hospital Capital Medical University Beijing China; ^5^ Department of Evidence‐based Medicine, Xuanwu Hospital Capital Medical University Beijing China

**Keywords:** cerebral venous thrombosis, inflammatory response, neurological injury, NLRP3 inflammasome, steroids

## Abstract

**Background:**

NLRP3 inflammasome‐related inflammation might play an important role in the pathophysiology of severe CVT. The use of steroids as anti‐inflammatory agents in improving severe CVT prognosis remains controversial.

**Methods:**

A total of 94 male Sprague–Dawley rats were used. We evaluated the dynamic and association between NLRP3 inflammasome in brain, blood, and CSF and severity in severe CVT rats and/or patients. We also explored the effect of steroids on NLRP3 activation, neurological injury, and CSF circulation disturbance after CVT in animals and/or patients.

**Results:**

In rats, compared with the sham group, NLRP3‐related factors rose on day 1, peaked on day 2 (NLRP3, Sham: 0.79 ± 0.22; day 2: 1.25 ± 0.08, *p* < 0.01; pro‐Caspase‐1, Sham: 0.58 ± 0.13, day 2: 1.20 ± 0.44, *p* < 0.05; GSDMD, Sham: 0.94 ± 0.22, day 2: 1.72 ± 0.46, *p* < 0.05; pro‐IL‐1β, Sham: 0.74 ± 0.15, day 2: 1.35 ± 0.09, *p* < 0.01), decreased on day 7 in rats (*n* = 4 per group). Thrombus (Sham: 0.00 ± 0.00, day 2: 3.44 ± 0.70, *p* < 0.0001), infarct size (Sham: 0.00 ± 0.00, day 2: 11.99 ± 6.26, *p* < 0.01) and neurological deficits appeared similar trend. In 50 patients, serum NLRP3 and IL‐6 levels correlated positively with NIHSS (*r* = 0.4273, *p* = 0.0020; *r* = 0.4938, *p* = 0.0029) and mRS (*r* = 0.5349, *p* = 0.0125; *r* = 0.6213, *p* = 0.026), while CSF IL‐6 correlated positively with mRS on admission (*r* = 0.5349, *p* = 0.0125). Compared with baseline, NLRP3 (0.36 (0.36, 0.36) vs. 0.41 (0.37, 0.84), *p* < 0.0001) and IL‐6 decreased (4.06 ± 1.48 vs. 12.03 ± 7.80, *p* < 0.05), accompanying by improvement of neurological deficits and CSF circulation (all *p* < 0.01) after steroids therapy in severe CVT patients at discharge and 3 months follow‐up. No significant steroid‐related adverse effects were observed.

**Conclusion:**

Short‐term steroid therapy may improve prognosis of severe CVT by suppressing NLRP3 inflammasome‐related inflammation.

## Graphical abstract

The NLRP3 inflammasome exhibited a strong correlation with neurological injury in severe CVT, both in rats and patients. The neuroprotective benefits of steroid treatment in severe CVT‐associated brain injury were possibly attributed to its ability to suppress NLRP3 inflammasome‐induced inflammation.

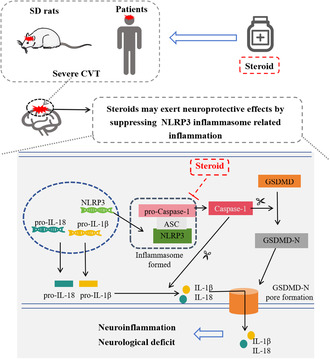

## 1 Introduction

Severe cerebral venous thrombosis (CVT) presents with severe manifestations, poor prognosis, and unclear mechanisms [1, 2]. Standard anticoagulation alone or in combination with endovascular treatments does not fully ameliorate patient outcomes [3], with a substantial long‐term poor prognosis rate (Modified Rankin Scale, mRS ≥ 2) of up to 32.8% [3]. Therefore, a renewed understanding of the pathophysiology will contribute to improving the prognosis of severe CVT.

Recent studies and our prior findings suggest that inflammation [4, 5, 6, 7, 8, 9, 10, 11], particularly the NLRP3 inflammasome [4, 5, 6, 7], plays a crucial role in regulating the pathogenesis of severe CVT. The NLRP3 inflammasome, a multiprotein complex comprising a sensor (NLRP3), an adaptor (ASC), and an executor (pro‐Caspase‐1), serves as the body's receptor for damage [12, 13]. Its canonical activation involves two distinct signals: an initiation signal that activates nuclear factor‐κB (NF‐κB), leading to the transcription of inflammatory factors NLRP3, pro‐IL‐1β, and pro‐IL‐18 [14, 15, 16]; and an activation signal triggered by danger‐associated molecular patterns, resulting in NLRP3 and pro‐Caspase‐1 oligomerization to form the NLRP3 inflammasome [14]. This triggers the cleavage of Caspase‐1, releasing mature IL‐1β and IL‐18 [12, 14], and cleaves gasdermin D (GSDMD), inducing pyroptosis. IL‐1β further enhances IL‐6 [17] and IL‐8 [18] expression, amplifying the inflammatory cascade and playing a role in cerebral ischemic injury [19]. The pyroptosis triggered by NLRP3 inflammasome‐mediated GSDMD is implicated in the pathological mechanisms of subarachnoid hemorrhage [20] and cerebral ischemia [21]. However, the precise role of NLRP3 inflammasome‐mediated inflammation in neurological injury in severe CVT and its potential as a therapeutic target remain to be fully elucidated.

Current anticoagulation and endovascular therapy for severe CVT lack the ability to selectively inhibit inflammatory cells and pathways [22]. Steroids, as broad‐spectrum anti‐inflammatory agents, activate their receptors to induce the expression of typical anti‐inflammatory proteins such as IL‐10 and IκBα, thereby inhibiting the transcription of inflammatory genes including IL‐6, IL‐1β, and NF‐κB [23]. Additionally, steroids suppress the activation of NLRP3 inflammasome [24]. In clinical practice, we combined steroids pulse therapy with standard anticoagulation in severe CVT, resulting in favorable clinical outcomes [25, 26]. Importantly, the latest Chinese guidelines [27] have incorporated our findings and upgraded the level of evidence for recommending steroids (Class IIb, Level of Evidence C) compared to previous guidelines [28, 29].

Therefore, this study is intended to investigate the association between NLRP3 inflammasome‐mediated inflammatory response and neurological injury in severe CVT from rat models and patients. Thereafter, we explored the potential and possible mechanism of steroids in improving the prognosis of severe CVT.

## 2 Materials and Methods

### 2.1 Animals

A total of 94 male Sprague–Dawley (SD) rats (280–320 g) were obtained from the Animal Experiment Center of Capital Medical University. Rats were housed in the humidity‐ and temperature‐controlled animal facility with a 12 h light–dark cycle for at least 3d before experiments. During this period, they had unrestricted access to food and water. All animal experiments were approved by the Animal Experiments and Experimental Animal Welfare Committee of Capital Medical University (AEEI‐2021‐241) and adhered strictly to the ARRIVE 2.0 guidelines for reporting animal research.

### 2.2 Experimental Group Design

#### 2.2.1 Experimental 1

Based on our previous and relevant CVT animal research [5, 30], we designed *n* = 4 or 5 per group Figure S1. Thirty‐two rats were randomized to 4 groups: a Sham group and three CVT subgroups (at 1, 2, and 7 d post‐CVT); brain tissue and peripheral serum were collected for Western blot (WB) and ELISA (*n* = 4 per group), respectively. Twenty rats were assigned to the Sham group and three CVT time points (1, 2, and 7 d post‐CVT, *n* = 5 per group) to assess thrombus load and cerebral infarct volume. Ten rats were assigned to the Sham group and CVT group (*n* = 5 per group) for continuous assessment (1, 2, 3, 5, and 7 d) of Neurological severity scores (NSS) and rotarod test.

#### 2.2.2 Experimental 2

A total of 32 rats were randomized to 2 groups: the dexamethasone phosphate solution (DXM) group and the normal saline (NS) group. Brain tissue and peripheral serum were collected for WB and ELISA (*n* = 4 per group) Figure S1. Eight rats were assigned to the DXM group and NS group (*n* = 4 per group) to assess thrombus load and cerebral infarct volume. Eight rats were assigned to the DXM group and NS group (*n* = 4 per group) for continuous assessment (1, 2, 3, 5, and 7 d) of NSS and rotarod test.

#### 2.2.3 Method, Dose, and Time of Administration

We chose DXM at a dose of 4 mg/kg body weight, once/day for 3 days, to be administered intraperitoneally immediately after modeling in severe CVT rats [31, 32]. In the control group, an equal volume of NS [32, 33] was injected intraperitoneally under the same conditions.

We performed inflammatory factors assessment, cerebral venous infarction, and thrombus load evaluation, and neurological dysfunction assessment (Supporting File S1).

#### 2.2.4 Clinical Research Design

The study was a retrospective study approved by the ethics committee of Xuanwu hospital ((2020)098), with informed consent from all patients. Fifty patients with newly diagnosed CVT by CT + CTV, MRI + MRV/MRBTI (MR Black‐Blood Thrombus Imaging), or DSA were included from 2018 to 2021 Figure S2. The thrombosis phase (acute, 0–7 days or subacute, 8–15 days) [10] was determined by history, MRI, and MRBTI. Patients of any gender and aged 14–80 years were included. Severe CVT was defined as combined cerebral venous infarction/hemorrhage, seizures or impaired consciousness, involvement of the straight sinus, and deep cerebral vein thrombosis; otherwise, it was defined as nonsevere [3, 4, 10].

Exclusion criteria included severe diseases with poor prognosis within 1 year (e.g., acute cerebral infarction, unstable angina, and uncontrollable asthma), immune‐related diseases (e.g., Behcet's disease, systemic lupus erythematosus), mechanical extraction of the venous sinus, and absolute contraindications to steroid use (e.g., uncontrollable infections, severe hypertension) [34].

Subsequently, we collected data and conducted a comprehensive assessment of neurological deficits (National Institute of Health stroke scale, NIHSS and Modified Rankin Scale, mRS), cerebrospinal fluid (CSF) circulation disorders (intracranial pressure (ICP) and optic papilla edema), and inflammatory factor detection.

All patients received standardized anticoagulation after diagnosis. Patients with severe CVT received steroid therapy short‐term pulsed treatment: 500 ‐mg methylprednisolone once a day, intravenous drip for 3 days, reduced to 80 mg once a day, intravenous drip for 3–5 days, and then changed to methylprednisolone/prednisone oral 1 mg/kg body weight, gradually reduced by 8 mg/10 mg per week until discontinued. Inflammatory factors and neurological function were evaluated after two weeks of steroid pulse (Supporting Information S1).

### 2.3 Statistical Analysis

GraphPad Prism 9 (GraphPad Software, La Jolla, CA) and SPSS 25.0 (SPSS, IBM, Armonk, NY, USA) were used for statistical analysis. The Shapiro–Wilk test was employed to assess the normality of the data. Measures conforming to normal distribution were expressed as mean ± standard deviation (SEM), while non‐normal distribution was expressed as median and interquartile range (IQR) (M (P25, P75)). Comparisons between two groups were performed by the Student's t test or rank sum test, and correlations between two variables were performed by the Pearson correlation test or Spearman correlation test. A Wilcoxon signed rank test or paired t test was used to compare indicators before and after short‐term steroid pulsed treatment. One‐way analysis of variance (ANOVA) was used for multi‐group analysis. A two‐tailed P < 0.05 was considered statistically significant.

## 3 Results

### 3.1 Basic Research

#### 3.1.1 The Levels of NLRP3 Inflammasome in Severe CVT Rats Increased Obviously in the Early Stage and Then Decreased

**FIGURE 1:**
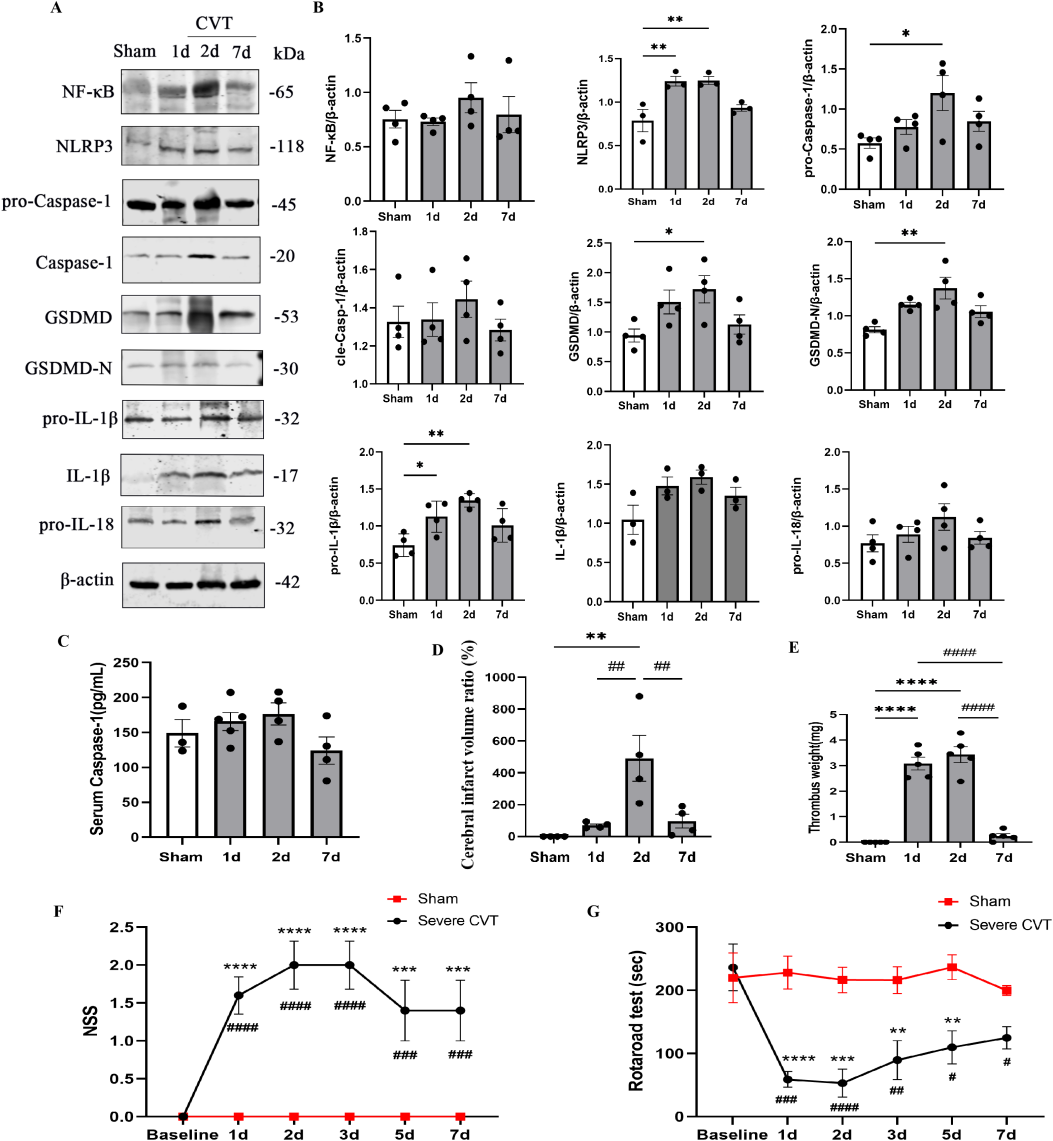
Dynamics of NLRP3 inflammasome, cerebral venous infarct volume, thrombus load, and neurological deficits after severe CVT. (A) Western blot assay for the temporal profiles of NLRP3 inflammasome‐associated molecular levels in the damaged cortex around SSS, including Sham, 1d, 2d, and 7d post‐CVT. (B) Quantitative analysis for Western blot. *n* = 4 rats per group. **p < 0.01, *p < 0.05. (C) Changes in peripheral blood Caspase‐1 levels detected by ELISA. *n* = 4 rats per group. (D) Quantitative analysis of cerebral infarct volume by TTC staining in the Sham group and CVT groups of rats at 1d, 2d, and 7d. *Indicates comparison of the group with the Sham group; #Indicates comparison of the group with day 2 after CVT, *n* = 4 rats per group, **p < 0.01, ##*p* < 0.01. (E) Quantification of thrombus load at 1d, 2d, and 7d after severe CVT. *Indicates comparison of the group with the Sham group; #Indicates comparison of the group with day 2 after CVT, *n* = 5 rats per group, ****p < 0.0001, ####*p* < 0.0001. Neurological deficits were assessed by (F) NSS and (G) rotarod test before operation (baseline) and 1d, 2d, 3d, 5d, and 7d after operation. *Indicates comparison of the group with the Sham group; #Indicates comparison of the group with baseline, *n* = 5 rats per group, ****p < 0.0001, ***p < 0.001, **p < 0.01, ####*p* < 0.0001, ###*p* < 0.001, ##*p* < 0.01, #*p* < 0.05. NSS, neurological severity. scores.

The results of WB revealed that compared to the Sham group, most molecules associated with the NLRP3 inflammasome started to increase on day 1, peaked on day 2, and then gradually decreased on day 7 after severe CVT (Figure 1A,B). The dynamics of inflammasome sensors NLRP3 (Sham: 0.79 ± 0.22, day 1: 1.24 ± 0.10, *p* < 0.01; day 2: 1.25 ± 0.08, *p* < 0.01) and downstream products pro‐IL‐1β (Sham: 0.74 ± 0.15, day 1: 1.13 ± 0.12, *p* < 0.05; day 2: 1.35 ± 0.09, *p* < 0.01) were particularly pronounced. Inflammasome executor pro‐Caspase‐1, pyroptosis effector GSDMD (Sham: 0.94 ± 0.22, day 2: 1.72 ± 0.46, *p* < 0.05), and GSDMD‐N (Sham: 0.81 ± 0.08, day 2: 1.37 ± 0.29, *p* < 0.05) demonstrated similar dynamics. Although the levels of upstream activator NF‐κB, activated Caspase‐1 (cle‐Caspase‐1), IL‐1β, and downstream pro‐IL‐18 remained relatively high until day 7, there were no statistical differences compared with the Sham group (Figure 1B). The expression of inflammatory factors in brain tissue of severe CVT represents part of the central inflammation, prompting us to investigate the periphery expression profile compared with that in the central. Therefore, we quantified Caspase‐1 levels in peripheral blood by ELISA. The results indicated that peripheral serum Caspase‐1 exhibited a similar trend as in the brain (Figure 1C).

#### 3.1.2 Neurological Injury Showed a Parallel Trend With the Changes of NLRP3 Inflammasome‐Related Molecules in Severe CVT Rats

Compared to the Sham group, white cerebral venous infarct tissue by TTC staining presented in the cortical area adjacent to the SSS in the severe CVT group (Figure S3). Cerebral infarction volume increased on day 1, reached a peak on day 2 (Sham: 0.00 ± 0.00, day 2: 11.99 ± 6.26, *p* < 0.01), and decreased significantly on day 7 (day 2:11.99 ± 6.26, day 7:1.84 ± 2.25, *p* < 0.01, Figure 1D). Similarly, thrombus load grew significantly on day 1 and peaked on day 2 after severe CVT (Sham: 0.00 ± 0.00, day 1: 3.08 ± 0.56, *p* < 0.0001; day 2: 3.44 ± 0.70, *p* < 0.0001, Figure 1E, Figure S3), experiencing a notable reduction by day 7 (day 7: 0.25 ± 0.20, day 1: 3.08 ± 0.56, *p* < 0.0001; day 2: 3.44 ± 0.70, *p* < 0.0001, Figure 1E).

Compared with the Sham group and baseline, the severe CVT group exhibited a significant increase in NSS at all postoperative time points. The NSS increased on day 1 peaked on days 2–3, and a gradual decrease on days 5–7 (Figure 1F). Likewise, severe CVT rats exhibited a significant decrease in rod retention duration during all postoperative periods. Specifically, the rod retention duration reached its lowest point on day l and remained low until day 2, before gradually increasing from days 3 to 7 after severe CVT (Figure 1G). In summary, the changes in neurological injury exhibited a more synchronized trend with expression of the NLRP3 inflammasome in rats with severe CVT.

#### 3.1.3 Steroid Treatment in Severe CVT Rats Seemed to Relieve Neuroinflammation and Neurological Deficits

**FIGURE 2:**
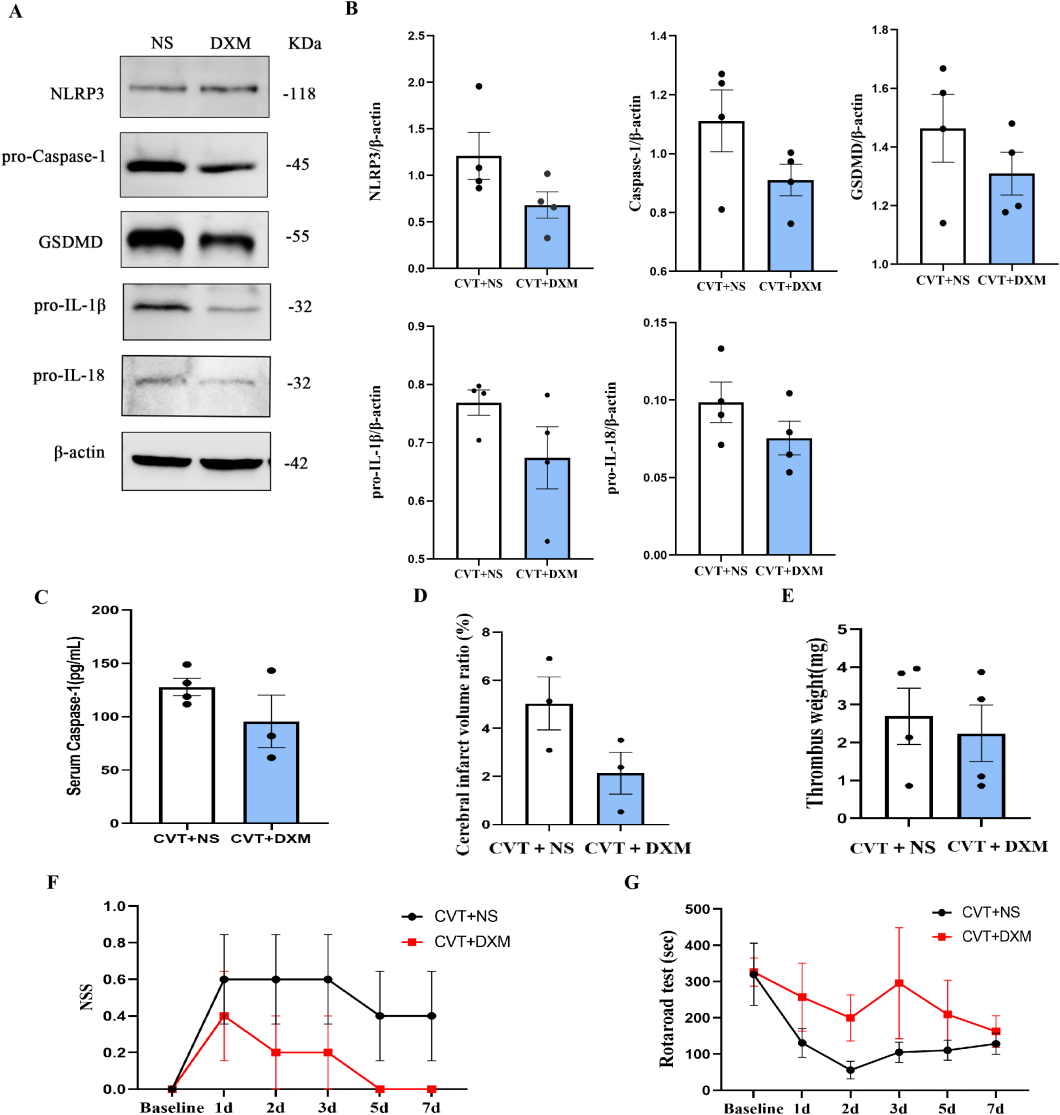
Effects of steroids (DXM) on neuroinflammation and neuronal injury after severe CVT. (A) Representative images of NLRP3 inflammasome‐associated molecular in the damaged cortex around SSS. (B) Quantitative analysis for Western blot. *n* = 4 rats per group. ( *C) ELISA* assay and quantitative analysis of Caspase‐1 in peripheral blood. (D) Quantitative analysis of cerebral infarct volume by TTC staining in diverse groups. *n* = 3 rats per group. (E) Quantification of thrombus in diverse groups. *n* = 4 rats per group. (F) Assessment of neurological deficits by NSS and (G) rotarod test in diverse groups. *n* = 5 rats per group. NSS, neurological severity scores.

On the 3rd day post‐CVT, brain levels of NLRP3, pro‐Caspase‐1, GSDMD, pro‐IL‐1β, and pro‐IL‐18 exhibited a decrease in rats in the DXM group compared to the NS group; however, statistical significance was not yet reached (Figure 2B). Peripheral serum Caspase‐1 showed parallel alterations (Figure 2C).

In addition, the DXM group exhibited a reduction in cerebral infarction on the 3rd day compared to the NS group (Figure 2D, Figure S4A). The use of steroids remains controversial due to their potential prothrombotic effects [35]. However, our study demonstrated that there was no significant increase in thrombus load in the DXM group compared to the NS group (2.24 ± 1.49 vs. 2.7 ± 0.48, *p* = 0.68, Figure 2E, Figure S4B). The rotarod test revealed that the NS group exhibited inferior motor function compared to those in the DXM group within one week, and motor function in the DXM group approached baseline levels by day 3 of continuous administration (Figure 2G). All NSS at one week were smaller in the DXM group than in the NS group (Figure 2F), but these have not yet reached statistical significance.

### 3.2 Clinical Research

#### 3.2.1 Patients

A total of 50 patients with acute/subacute CVT were recruited from February 2018 to December 2021, and they were categorized into severe (*n* = 25) and nonsevere (*n* = 25) groups based on criteria. The specific demographics are presented in Table 1. All patients were followed up at 3 months of discharge.

**TABLE 1:** Demographics of the acute/subacute CVT patients.

Variables (*N*)	CVT patients(*N* = 50)
Females, *N* (%)	23 (46)
Age, Mean ± SEM	39.84 ± 14.35
Risk factors, *N* (%)	
Estrogen use (% female)	6 (26)
Pregnancy or puerperium (% female)	3 (13)
Previous history of venous thrombosis (%)	3 (6)
Protein S deficiency (%)	2 (4)
Protein C deficiency (%)	1 (2)
Antithrombin III deficiency (%)	2 (4)
NIHSS scale on admission, M (P25, P75)	0.00 (0.00, 6.25)
mRS scale on admission, M (P25, P75)	0.00 (0.00, 4.00)

#### 3.2.2 Intense Inflammasome‐Associated Inflammation Correlated With the Severity of CVT

**FIGURE 3:**
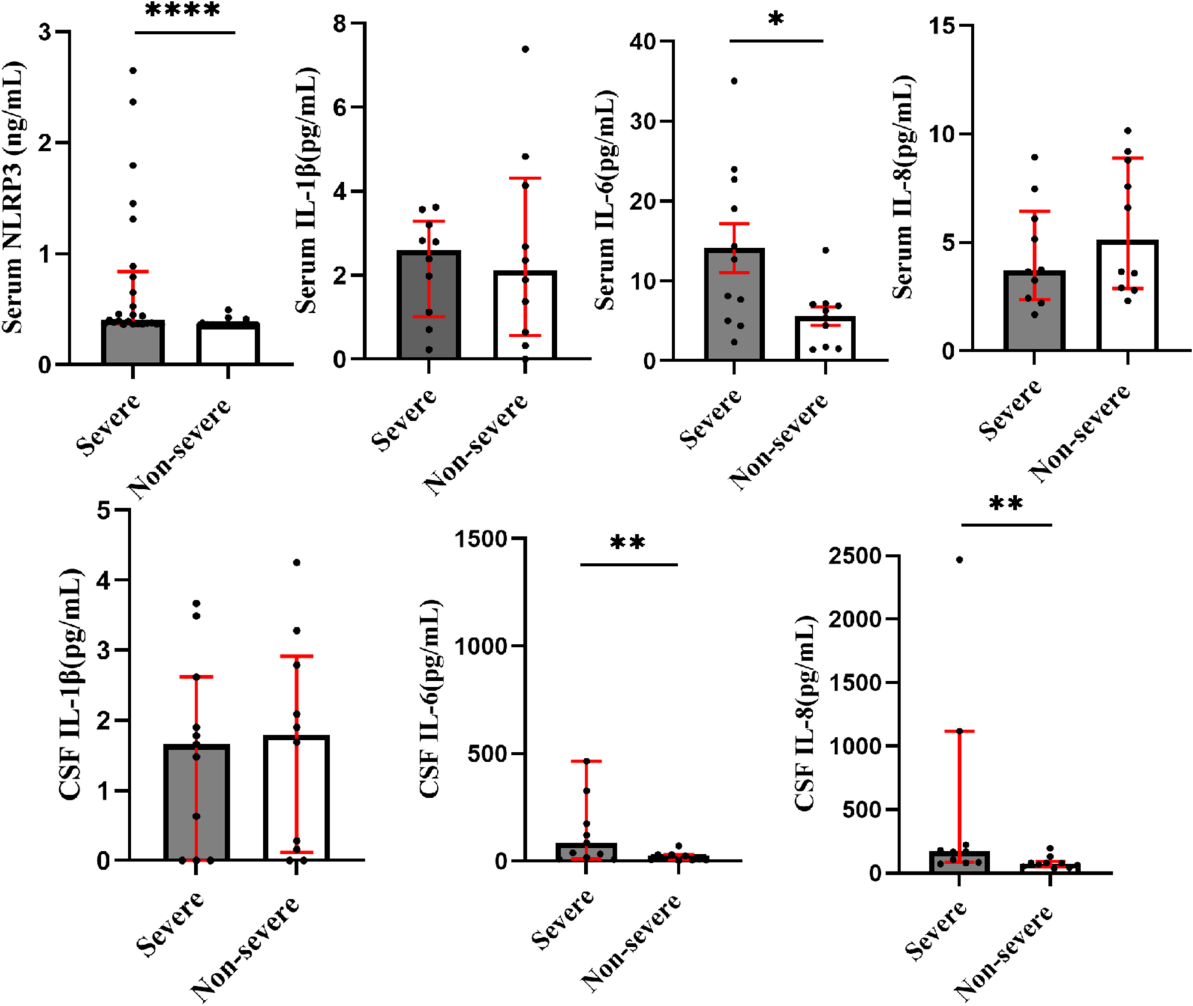
Comparison of NLRP3 inflammasome‐associated molecular levels in patients with acute/subacute CVT. Detection of NLRP3 and IL‐1β, IL‐6, and IL‐8 using ELISA and CBA assays, respectively. ****p < 0.0001, **p < 0.01, *p < 0.05. CSF, cerebrospinal fluid.

On admission, serum levels of NLRP3 (0.41(0.37, 0.84) vs. 0.36 (0.35, 0.36), *p* < 0.0001, Figure 3) and IL‐6 (14.13 ± 10.13 vs. 5.58 ± 3.73, *p* < 0.05, Figure 3) were significantly elevated in severe CVT patients (*n* = 11) compared to nonsevere CVT patients (*n* = 10). No significant differences were observed in serum IL‐1β and IL‐8 between the two groups. In CSF, a significant increase was observed in IL‐6 (102.36 (29.00, 361.50) vs. 9.67 (4.28, 31.09), *p* < 0.01) and IL‐8 (174.00 (100.30, 1455.01) vs. 73.03 (49.56, 92.81), *p* < 0.01) in the severe group compared to the nonsevere group, while no significant difference was found in IL‐1β between the two groups (Figure 3).

**FIGURE 4:**
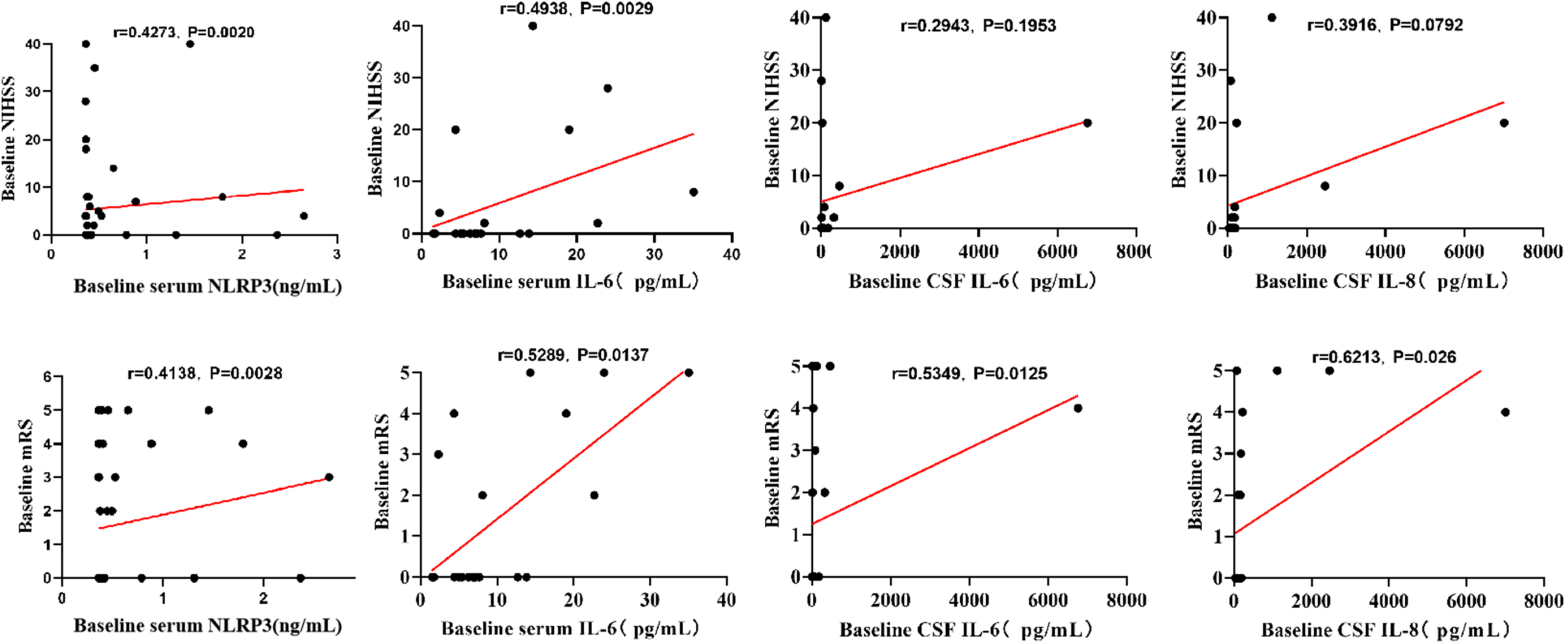
The correlation between NLRP3 inflammasome‐associated molecules and the severity of CVT. NIHSS: National Institutes of Health Stroke Score, mRS; Modified Rankin Scale, CSF. cerebrospinal fluid.

The correlation analysis revealed a positive association between serum NLRP3 and IL‐6 levels with NIHSS (*r* = 0.4273, *p* = 0.0020; *r* = 0.4938, *p* = 0.0029, Figure 4) and mRS (*r* = 0.4138, *p* = 0.0028; *r* = 0.5289, *p* = 0.0137, Figure 4). Meanwhile, CSF IL‐6 and IL‐8 were positively correlated with mRS (*r* = 0.5349, *p* = 0.0125; *r* = 0.6213, *p* = 0.026, Figure 4) on admission. The results indicated that NLRP3 inflammasome‐related inflammation may be associated with the severity of CVT.

#### 3.2.3 Steroid Therapy Might Improve the Prognosis of Severe CVT by Mitigating the NLRP3 Inflammasome‐Related Inflammation

**FIGURE 5:**
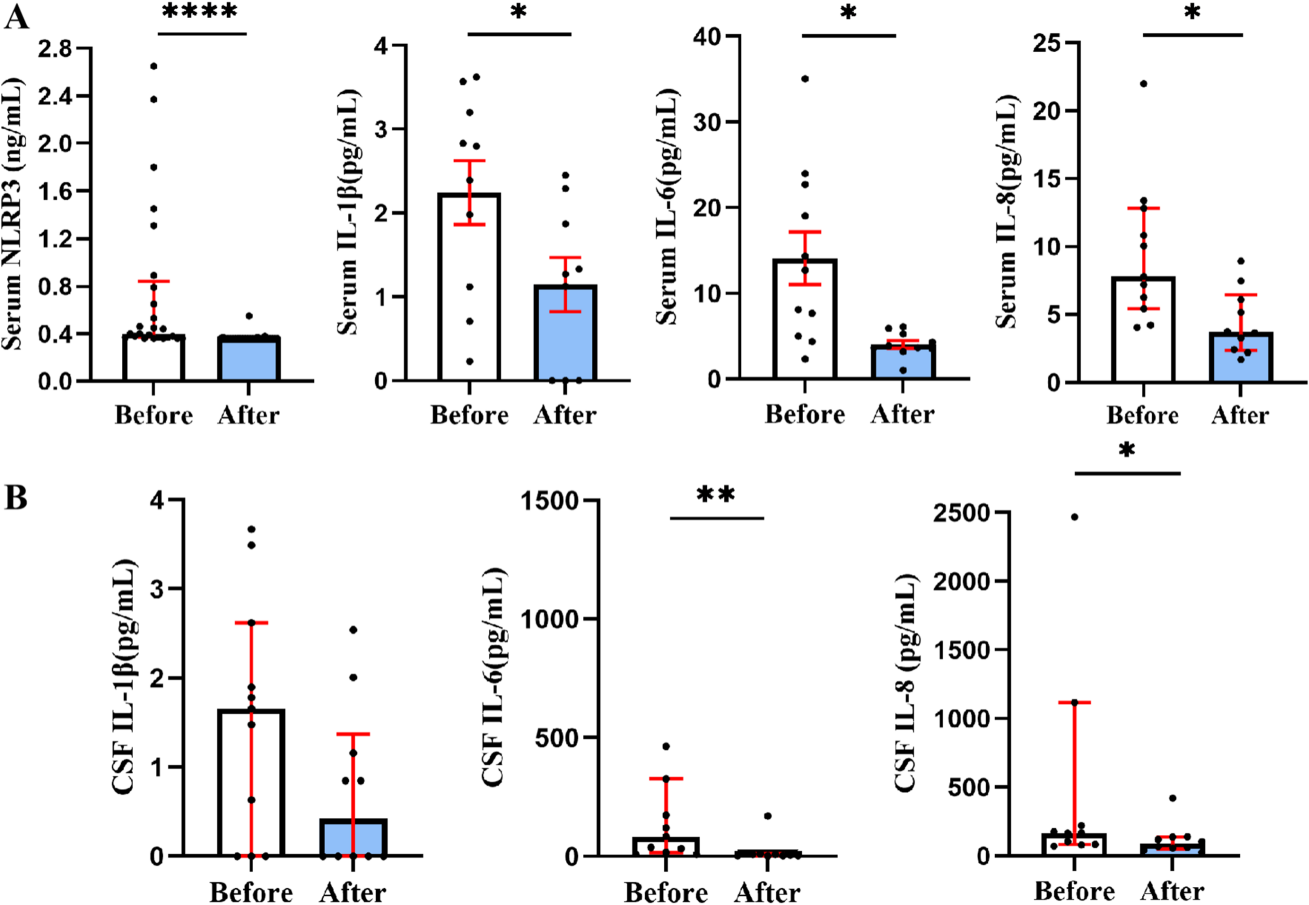
Significant reduction of NLRP3 inflammasome‐associated molecules in severe CVT after steroid pulse therapy. Detection of A. serum and B. CSF NLRP3 and IL‐1β, IL‐6, and IL‐8 using ELISA and CBA assays, respectively. ****p < 0.0001, **p < 0.01, *p < 0.05. Normally distributed bars represent data as mean ± standard error, and non‐normally distributed bars represent median ± interquartile range. CSF, cerebrospinal fluid.

First, we assessed the effect of steroids on the inflammation by combining steroid pulse with standard anticoagulation in severe CVT. After 2 weeks of steroid pulse (500 mg methylprednisolone i.v. drip for 3–5 days), we observed a significant reduction level in serum NLRP3 (0.36 (0.36, 0.36) vs. 0.41 (0.37, 0.84), *p* < 0.0001, Figure 5A), as well as decreased IL‐1β (1.21 ± 0.96 vs. 2.27 ± 1.33, *p* < 0.05), IL‐6 (4.06 ± 1.48 vs. 12.03 ± 7.80, *p* < 0.05) and IL‐8 (3.70 (2.37, 6.44) vs. 7.81 (5.43, 12.83), *p* < 0.05, Figure 5A) compared to the baseline. Additionally, there were significant decreases in the levels of CSF IL‐1β, IL‐6 (6.35 (3.21, 12.83) vs. 84.43 (16.74, 327.22), *p* < 0.01), and IL‐8 (88.36 (53.84, 138.21) vs. 69.63 (84.85, 1117.17), *p* < 0.05, Figure 5B). These findings indicated that both peripheral and central inflammatory responses showed improvement following steroid pulse.

Furthermore, we noted a significant reduction in NIHSS at discharge (*p* < 0.01, Table 2), which further declined by 3 months postdischarge (*p* < 0.001, Table 2). Similarly, mRS demonstrated a trend toward improvement at discharge (*p* = 0.056, Table 2) that became statistically significant by 3 months (*p* < 0.001, Table 2). Moreover, the ICP was significantly reduced at discharge compared to baseline (*p* < 0.01, Table 2), while the Frisén grade showed a significant decline by 3 months postdischarge (*p* < 0.05, Table 2), indicating an improvement in CSF circulation. These findings highlight the therapeutic potential of steroids in reversing neurological deficits and CSF circulation disorders in CVT patients.

**TABLE 2:** Comparisons of neurological deficits and increased intracranial pressure in severe CVT patients at admission, discharge, and 3 months after discharge.

Evaluation indicators	On admission (*n* = 25)	At discharge (*n* = 25)	At 3 months after discharge (*n* = 25)
NIHSS	6.00 (2.00, 19.00)	1.00 (0.00, 2.00)**	0.00 (0.00, 0.50)***
mRS	4.00 (2.00, 5.00)	1.00 (0.00, 2.00)	0.00 (0.00, 1.00)***
Intracranial pressure (mmH2O)	230.00 (175.00, 330.00)	187.50 (161.25, 238.75)**	—
Frisén grade	0.00 (0.00, 2.00)	—	0.00 (0.00, 0.00)*

* Indicates comparison with admission, ***P<0.001, **P<0.01, *P<0.05.

#### 3.2.4 No Steroid‐Related Serious Adverse Reactions in Patients With Severe CVT

In our observation and follow‐up, none of the 25 severe patients experienced recurrence of thrombosis or venous thrombosis in other anatomical locations during steroid treatment and 3 months after discharge. No significant adverse reactions related to steroids were observed, including spontaneous bone fracture or osteonecrosis, inducing or aggravating infections, and inducing or exacerbating gastroduodenal ulcers.

## 4 Discussion

This study is the first to investigate the impact of steroids on inflammation in severe CVT from both animal and clinical perspectives. We observed a pronounced upregulation of NLRP3 inflammasome‐related molecules in both experimental rats and patients with severe CVT, which strongly correlated with neurological damage. Notably, steroids may exert neuroprotective effects by inhibiting NLRP3 inflammasome‐related factors. Our findings highlighted the therapeutic merits of steroids in managing severe CVT, as evidenced by a notable reduction in cerebral infarct volumes, improved motor function among rats, and enhanced neurological outcomes (NIHSS and mRS), accompanied by a decrease in intracranial hypertension in patients. Importantly, no major adverse effects of steroids were observed.

It is widely recognized that the occurrence of severe CVT can be attributed to venous return disorder [26, 36, 37]. However, endovascular treatment for improving venous return in CVT patients did not improve functional outcomes [3]. Recent evidence links CVT pathology to NLRP3 inflammasome‐driven inflammation [4, 5, 6, 7, 8, 9, 10, 11]. Our results indicated NLRP3 inflammasome activation in both severe CVT rats and patients in the short term, aligning with previous animal studies [4, 5]. The activation of the NLRP3 inflammasome is often accompanied by pyroptosis, an underappreciated mechanism contributing to neurological injury following CVT [12, 14], which was investigated in our animal studies. We observed a temporal profile of pyroptosis factor GSDMD and GSDMD‐N in the brain of severe CVT rats as that of the NLRP3 inflammasome, consistent with previous research [5, 6]. These results strongly suggested a significant role for NLRP3 inflammasome activation and subsequent pyroptosis in the pathophysiology of severe CVT. Importantly, we observed a parallel increase in serum Caspase‐1 and brain levels in severe CVT rats. In severe CVT patients, elevated levels of serum NLRP3, IL‐6 along with CSF IL‐6 were observed on admission. These findings, which have not been previously reported, suggest that alterations in peripheral inflammatory factors may partially reflect central inflammation. On the other hand, in rats, neurological impairments, including cerebral infarction, thrombus load, and motor dysfunction, progressively worsened from day 1, peaked on day 2, and significantly improved by day 7. In patients, the level of NLRP3 inflammasome‐related factors in serum and CSF were positively correlated with NIHSS and mRS on admission. These results further support the significant association between post‐CVT NLRP3 inflammasome and neurological injury from both basic and clinical perspectives, suggesting that inhibiting NLRP3 inflammasome‐related inflammation may represent early intervention in severe CVT‐induced neurological injury.

Inhibition in the upstream of the NLRP3 inflammasome results in improvement in motor dysfunction in CVT mice [6]. In addition to targeting specific factors, broad‐spectrum anti‐inflammatory drugs, steroids, can modulate the neurosteroid receptor pathway, influencing neuroinflammation and thereby exerting neuroprotective effects poststroke [38, 39]. We recently explored the combination of steroids and anticoagulants for managing severe CVT patients, revealing a significant reduction in inflammatory markers in serum and CSF, accompanied by notable improvements in prognosis following steroid administration [24]. Notably, the latest Chinese guidelines [27] referenced our findings [26] and incorporated recommendations for the administration of steroids for severe CVT. Therefore, steroid use in severe CVT should not be determined prematurely, and further studies are still needed.

In this study, we observed a decrease in the expression of NLRP3 inflammasome factors in the brains and serum of rats treated with DXM compared with the NS group. Clinical results suggested a significant decrease in NLRP3 inflammasome molecules in serum and CSF of severe CVT patients following a steroid pulse. Moreover, rats treated with DXM exhibited improvements in brain injury and motor dysfunction. Notably, rats' motor function improved with DXM injection but later declined, probably attributed to DXM cessation. Nevertheless, this decline remained superior compared to the NS group. In patients, we employed the NIHSS and mRS to assess neurological damage; we also employed, for the first time, ICP and optic papillae edema assessment (Frisén grade) to evaluate CSF circulation disorders, thereby enabling a comprehensive prognosis assessment of CVT. The results revealed steroids pulse therapy led to a decrease in NIHSS, mRS, and ICP at discharge in severe CVT. There was a further reduction in NIHSS, mRS, and Frisén grade at 3 months postdischarge. In summary, steroids may achieve neuroprotection by inhibiting NLRP3 inflammasome factors in severe CVT.

The use of steroids in severe CVT is controversial primarily due to potential prothrombotic effects [35, 39] and adverse effects [34]. We found no significant increase in thrombus in rats with DXM. Similarly, during hospitalization and follow‐up of severe CVT patients, we did not observe any prothrombotic events with steroids, probably due to the inhibitory effect of steroids on the reciprocal promotion between inflammation and thrombus [11, 22]. Moreover, we also implemented a combination therapy of anticoagulation and steroids, which effectively mitigated the prothrombotic effect of steroids. Our follow‐up results showed no thrombus recurrence or significant steroid‐related adverse effects in severe CVT, indicating long‐term prognosis may not be negatively impacted using steroids. This may be attributed to our synergistic approach combining steroids with adjuvant therapies such as anticoagulants, gastric acid inhibitors, and calcium supplementation [26]. These results indicated that short‐term use of steroids for acute/subacute severe CVT appears to be safe and effective.

Our study had several strengths. First, we innovatively conduct a preliminary study to explore whether steroids could improve neurological injury by inhibiting the expression of NLRP3 inflammasome‐related factors from animal and clinical perspectives. Second, our clinical study incorporated metrics for evaluating CSF circulation disorders, representing the first instance of their inclusion in such research. The comprehensive assessment further enhances the prognostic evaluation of severe CVT and bolsters the credibility of our findings. However, there are certain limitations in this study. First, different gradient concentrations of DXM in rats are also needed to further validate our findings. Second, as a small retrospective study, our clinical study lacks a control group, which may cause some bias. We intend to design high‐quality prospective randomized controlled studies to further validate the efficacy and safety of steroids in patients with acute/subacute severe CVT.

## 5 Conclusion

In both rats and patients, the NLRP3 inflammasome‐associated inflammatory response may potentially contribute to neurological damage in severe CVT. The short‐term use of steroids may help mitigate neurological injury in severe CVT by suppressing the expression of NLRP3 inflammasome‐related molecules.

## Author Contributions

S.H. performed the CVT model, Western blotting, ELISA, and CBA. S.H. and Y.G. performed neurobehavioral test. S.H. and J.D. were involved in gaining ethical approval. S.H., J.D., and L.H. were involved in sample collection. S.H. and C.W. prepared the figures and data analysis. S.H., J.D., and X.J. conceived and designed experiments and interpreted results. J.L., H.Z., Y.L., and G.L. contributed to the refinement of the experimental protocol. S.H. wrote the first draft of the manuscript. J.D. revised the draft of the manuscript. All authors read and approved the final version of the manuscript.

## Ethics Statement

All experimental procedures were approved by the Capital Medical University Ethics Committee and were performed in conformity with the guidelines of the National Institutes of Health on the care and use of animals.

## Consent

The authors have nothing to report.

## Conflicts of Interest

Prof. Yumin Luo and Dr. Jia Liu are Editorial Board members of CNS Neuroscience and Therapeutics and co‐authors of this article. To minimize bias, they were excluded from all editorial decision‐making related to the acceptance of this article for publication.

## Supporting information


Figure S1.
Figure S2.
Figure S3.
Figure S4.

## Data Availability

The data that support the findings of this study are available from the corresponding author upon reasonable request.
