# Supplementary material for: Steroids' Neuroprotective Potential in Severe Cerebral Venous Thrombosis: Experimental and Clinical Exploration of NLRP3 Inflammasome Inhibition
Source: CNS Neurosci Ther. 2024 Nov 17;30(11):e70125. doi: 10.1111/cns.70125 (PMC11570549; doi:10.1111/cns.70125)
Supplement: Supplementary file 1 — Figure S1. Figure S2. Figure S3. Figure S4. [file CNS-30-e70125-s001.docx]

**Supplementary Information**

**Supplementary File 1: Materials and Methods**

**Supplementary Figure 1: Animal experimental design and grouping.**

**Supplementary Figure 2: Clinical design and patients grouping.**

**Supplementary Figure 3: TTC staining and thrombus images of Sham and CVT groups.**

**Supplementary Figure 4: TTC staining and thrombus images of DXM and NS groups.**

**Reference**

**Supplementary File 1: Materials and Methods**

**Animal research**

**The novel severe CVT rat model**

A novel severe CVT model was induced in rats using semi-ligation combined with ferric chloride and thrombin as we previously described (1). In brief, rats were anesthetized with 5% enflurane in the air by facemask. A high-speed dental drill was used to create a longitudinal cranial window (10×4 mm) between the rat brema and lambda, exposing the superior sagittal sinus (SSS) and bilateral cortex. Subsequently, the rostral and caudal of the SSS were semi-ligated using 8-0 polyamide sutures. A section of 3-0 silk thread dipped in 40% ferric chloride was then placed over the SSS surface for 5 min in the dark. Next, thrombin was injected into the sinus cavity of the ligated segment within 1 minute using a microsyringe. The Sham-operated animals underwent the same anesthesia and cranial window fenestration.

**Inflammatory factors assessment**

**Western Blot**

The rats were sacrificed, and the SSS and the surrounding 1/3 of brain tissue were collected and homogenized using an ultrasonic pulverizer. The steps were similar to previous studies (2). Briefly, the main steps of protein extraction, concentration detection; electrophoresis and membrane transfer, incubation of primary antibodies (NLRP3, 1:1000, 19771-1-AP, Proteintech, China; Caspase-1, 1:1000, ab179515, Abcam, UK; GSDMD, 1:1000, ab219800, Abcam; IL-1β, 1:1000, GTX74034, Gen Tex, USA; IL-18 ,1:1000, ab191860, Abcam; NF-κB, 1:1000, GTX107678, Gene Tex; and β-actin, 1:1000, ab8224, Abcam) and secondary antibodies (Goat anti-Rabbit IgG Secondary Antibody, 1:10000, 926-68071, Licor, USA; Goat anti-Mouse IgG Secondary Antibody, 1:10000, 926-32210, Licor) were performed. Finally, scan target protein bands using Odyssey Infrared Fluorescence Imaging System and analyze semi-quantitatively with Image J.

**Enzyme- linked immunosorbent assay (ELISA)**

After anesthetizing rats, blood was drawn from the inferior vena cava, left at room temperature for 2h, centrifuged at 3000 rpm for 15min, and the supernatant was collected as serum. Serum was tested by ELISA using Caspase-1 ELISA kit (E-EL-R0371c, Elabscience, China).

**Cerebral venous infarction volume and thrombus load assessment**

Rats's brain tissue was sliced into 2-mm sections rostrocaudally. These slices were then incubated in 2% 2,3,5-triphenyl tetrazolium chloride (TTC, T8170, Solarbio, China) solution at 37°C for 20 minutes in the dark. The cerebral infarct volume ratio = (sum of white infarction area in each section)/(sum of brain slice area in each section) × 100%. The rats' skulls were opened to visually observe thrombus load after saline perfusion. The thrombus was later isolated, dried, and weighed for quantitative analysis (1).

**Neurological dysfunction evaluation**

The neurological dysfunction function was evaluated using NSS and the rotarod test. The NSS score ranges from 0-3 (3). For the rotarod test, rats were trained for 3 consecutive days, 3 times daily, prior to surgery. The third day's performance was considered the baseline. The rotarod speed was gradually accelerated from 4 to 40 rpm in 300s. All rats were tested on days 1,2,3,5 and 7 post-modeling (1).

**Clinical research**

**Inflammasome product assay**

Peripheral venous blood and/or CSF were collected from all CVT patients on admission; and again 2 weeks after steroid pulse treatment in severe CVT patients. Samples were centrifuged (100g for 20 min), and serum NLRP3 and IL-1β levels measured using ELISA kit and Cytometric bead array, respectively. Assays were performed using the BD FACSCanto II flow cytometer and FCAP Array™ v 3.0.1 software.

**Supplementary Figure 1: Animal experimental design and grouping.**

**
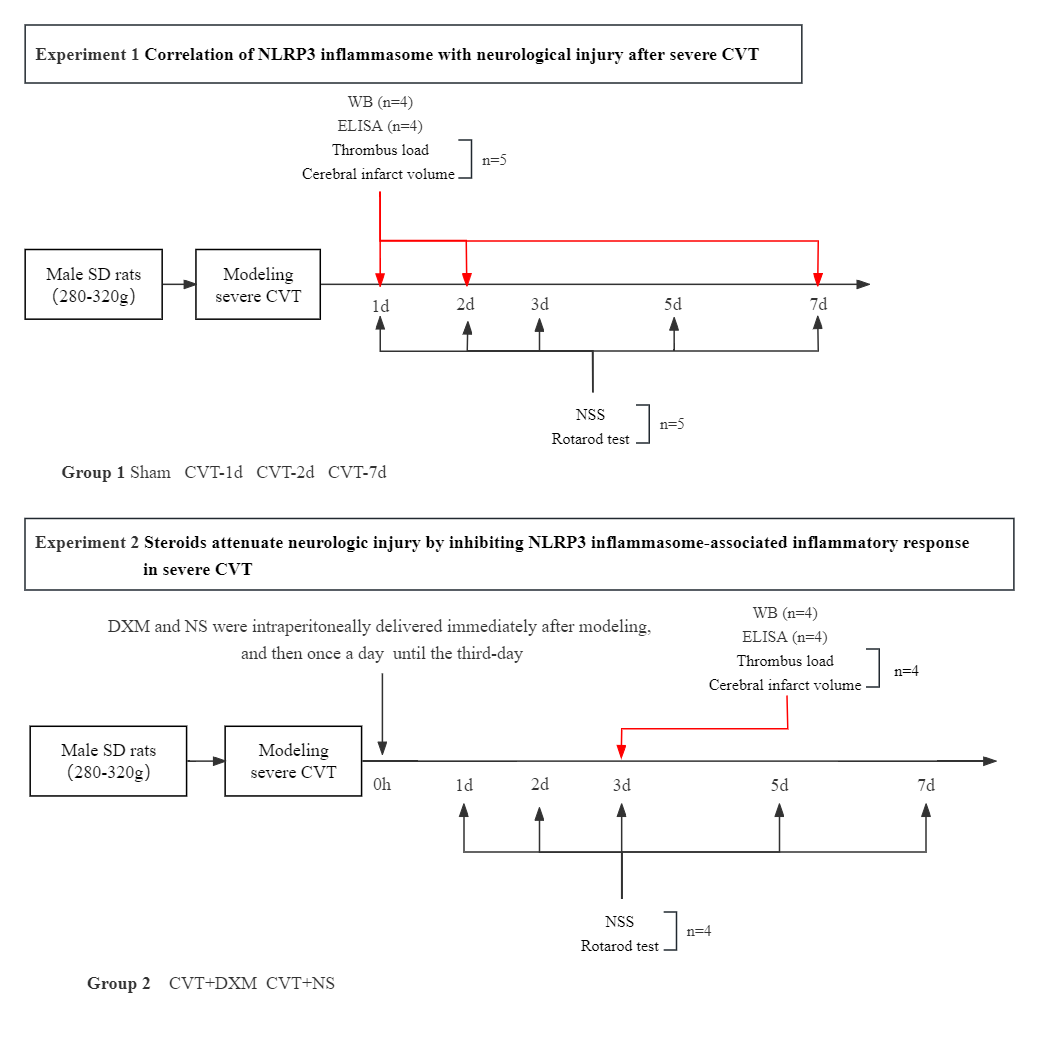
**

**Fig. S1. Animal experimental design and grouping.** CVT, cerebral venous thrombosis; WB, western blot; ELISA, Enzyme linked immunosorbent assay; NSS, neurological severity scores; NS, normal saline; DXM, dexamethasone.

**Supplementary Figure 2: Clinical design and patients grouping.**

**
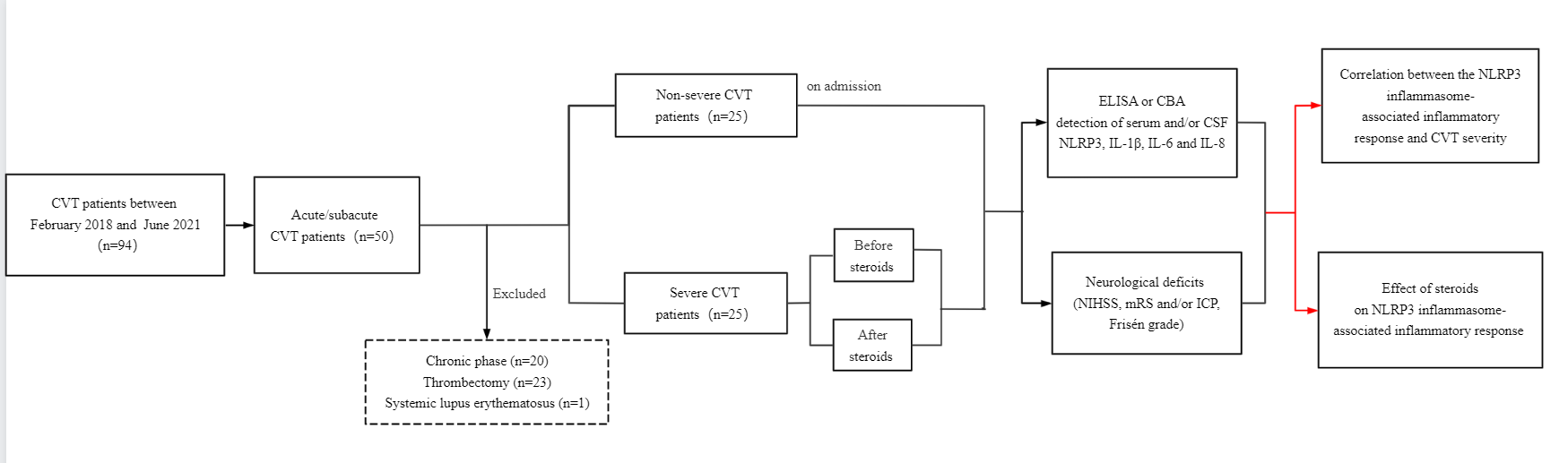
**

**Fig. S2. Clinical design and patients grouping.** CVT, cerebral venous thrombosis; ELISA, Enzyme linked immunosorbent assay; CBA, Cytometric bead array; NIHSS, National Institutes of Health Stroke Score; mRS, modified Rankin Scale; ICP:intracranial pressure.

**Supplementary Figure 3: TTC staining and thrombus images of Sham and CVT groups.**

**
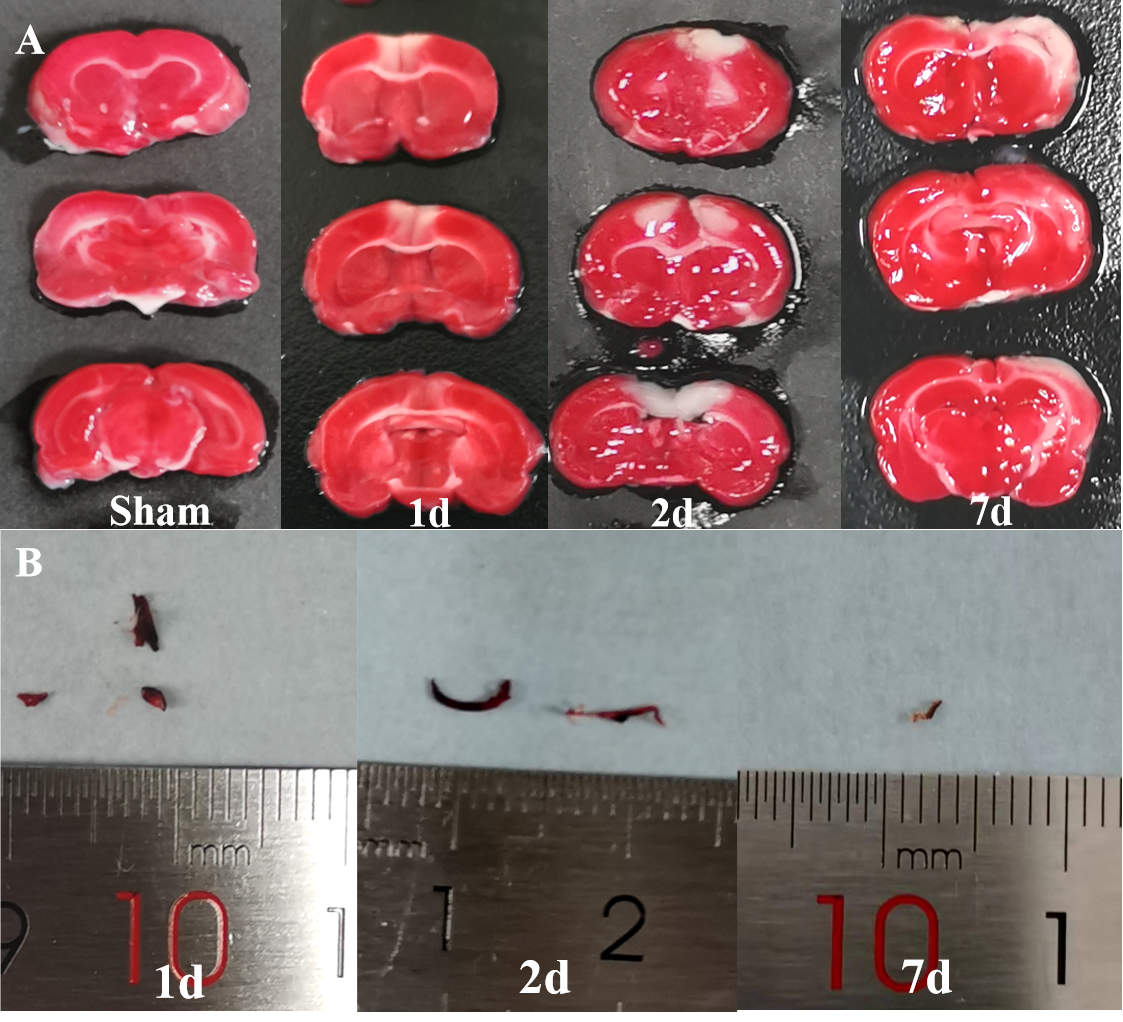
**

**Fig. S3.** A. TTC staining of Sham group and CVT subgroups (1d, 2d and 7d). White represents cerebral venous infarction. B. Thrombus images in Sham group and CVT subgroup (1d, 2d and 7d).**Supplementary Figure 4: TTC staining and thrombus images of DXM and NS groups.**

**
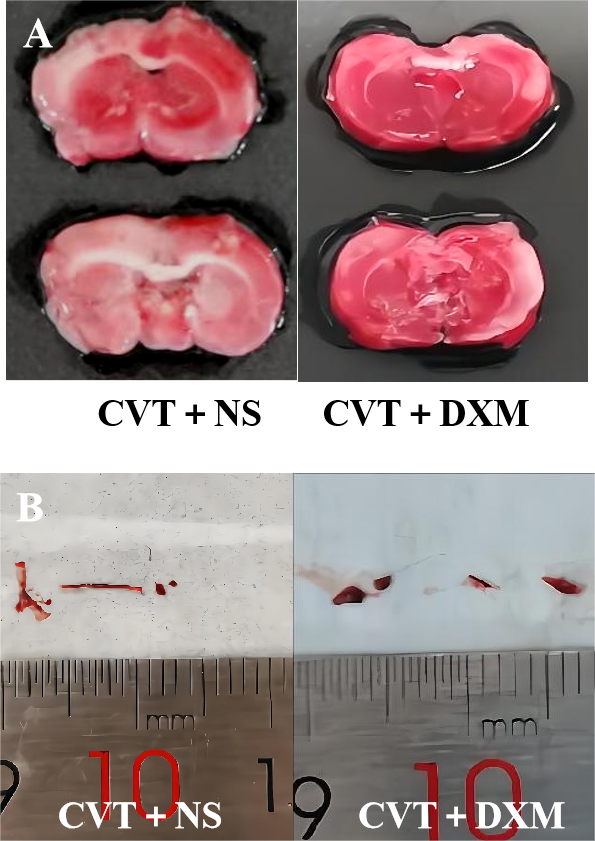
**

**Fig. S4.** A. TTC staining of CVT+NS group and CVT+DXM group. White represents cerebral venous infarction. B. Thrombus images in CVT+NS group and CVT+DXM group.

**References:**

1. Xiao L, Ji X, Zhao H, Luo Y, Hu S, Zhao T, Hu Z, Duan J. A novel severe cerebral venous thrombosis rat model based on semi-ligation combined with ferric chloride and thrombin. *Cns Neurosci Ther*. 2022;28:2129-2140

2. Ding R, Li H, Liu Y, Ou W, Zhang X, Chai H, Huang X, Yang W, Wang Q. Activating cgas-sting axis contributes to neuroinflammation in cvst mouse model and induces inflammasome activation and microglia pyroptosis. *J Neuroinflammation*. 2022;19:137

3. Srivastava AK, Kalita J, Dohare P, Ray M, Misra UK. Studies of free radical generation by neurons in a rat model of cerebral venous sinus thrombosis. *Neurosci Lett*. 2009;450:127-131
